# Hsp90 mutants with distinct defects provide novel insights into cochaperone regulation of the folding cycle

**DOI:** 10.1371/journal.pgen.1010772

**Published:** 2023-05-25

**Authors:** Rebecca Mercier, Danielle Yama, Paul LaPointe, Jill L. Johnson

**Affiliations:** 1 Department of Cell Biology, Faculty of Medicine and Dentistry, the University of Alberta, Edmonton, Alberta, Canada; 2 Department of Biological Sciences, University of Idaho, Moscow, Idaho, United States of America; University of Bristol, UNITED KINGDOM

## Abstract

Molecular chaperones play a key role in maintaining proteostasis and cellular health. The abundant, essential, cytosolic Hsp90 (Heat shock protein, 90 kDa) facilitates the folding and activation of hundreds of newly synthesized or misfolded client proteins in an ATP-dependent folding pathway. In a simplified model, Hsp70 first helps load client onto Hsp90, ATP binding results in conformational changes in Hsp90 that result in the closed complex, and then less defined events result in nucleotide hydrolysis, client release and return to the open state. Cochaperones bind and assist Hsp90 during this process. We previously identified a series of yeast Hsp90 mutants that appear to disrupt either the ‘loading’, ‘closing’ or ‘reopening’ events, and showed that the mutants had differing effects on activity of some clients. Here we used those mutants to dissect Hsp90 and cochaperone interactions. Overexpression or deletion of *HCH1* had dramatically opposing effects on the growth of cells expressing different mutants, with a phenotypic shift coinciding with formation of the closed conformation. Hch1 appears to destabilize Hsp90-nucleotide interaction, hindering formation of the closed conformation, whereas Cpr6 counters the effects of Hch1 by stabilizing the closed conformation. Hch1 and the homologous Aha1 share some functions, but the role of Hch1 in inhibiting progression through the early stages of the folding cycle is unique. Sensitivity to the Hsp90 inhibitor NVP-AUY922 also correlates with the conformational cycle, with mutants defective in the loading phase being most sensitive and those defective in the reopening phase being most resistant to the drug. Overall, our results indicate that the timing of transition into and out of the closed conformation is tightly regulated by cochaperones. Further analysis will help elucidate additional steps required for progression through the Hsp90 folding cycle and may lead to new strategies for modulating Hsp90 function.

## Author summary

Hsp90 undergoes a series of complex conformational changes as it progresses through the ATP-dependent folding cycle. Using a series of yeast Hsp90 mutants defective at distinct steps, we demonstrate that the Hch1, Aha1 and Cpr6 cochaperones tightly regulate Hsp90 function in vivo, supporting a role as conformational pacemakers of Hsp90. In particular, our results suggest that Hch1 negatively regulates progression through the cycle by reducing Hsp90 interaction with ATP. We also show that sensitivity to Hsp90 inhibitors also correlates with conformation. Our results provide evidence that timing of Hsp90 conformational changes, rather than ATPase activity, is critical, and that cochaperones play an active role in regulating this process.

## Introduction

Hsp90 is an abundant, essential molecular chaperone that promotes the folding, activation or maturation of an estimated 10–15% of the yeast and human proteome [1,2]. Hsp90 functions as a dimer and is highly conserved from bacteria to humans. Human cells contain four genes encoding Hsp90, which includes two isoforms of cytosolic Hsp90, mitochondrial TRAP1 and ER localized GRP94 [3,4]. Hsp90 clients play critical roles in cell signaling pathways, including multiple proteins involved in carcinogenesis and neurodegeneration [5–7]. Hsp90 inhibitors that bind the ATP-binding pocket have been developed as anti-cancer agents. NYP-AUY922 (luminespib) has anti-tumor, anti-angiogenesis and anti-metastasis activity and has been used in multiple studies to determine the impacts of Hsp90 inhibition [8,9]. While these drugs are effective, negative side effects have limited their clinical use to date [6,10]. A better understanding of the factors that dictate client folding may enable development of more specific inhibitors with fewer side effects.

Cytosolic Hsp90 assists protein folding in a multistep pathway in which client protein progresses through a series of complexes containing Hsp90 and associated cochaperone proteins [11–15]. There are at least twelve cochaperones in *Saccharomyces cerevisiae* and approximately 50 in human cells [16–18]. Cochaperones target clients to Hsp90, regulate the ATPase activity of Hsp90, and mediate conformational changes in Hsp90 as it progresses through the cycle [3]. Deletion of some cochaperones in yeast affects activity of multiple clients, suggesting they are part of the core folding cycle, while others may be part of more select networks. Although early studies focused mostly on how cochaperones regulate Hsp90 ATPase activity, recent studies indicate that cochaperones directly contact clients and influence client conformation [11,12,15,17]. In addition, changes in the relative balance of cochaperones proteins has dramatic effects on client fate [19,20].

Yeast and human Hsp90 have similar interactions with cochaperones, a common structure, and undergo similar nucleotide-induced conformational changes, but there are differences in conformational dynamics, ATPase rates, and cohort of available cochaperones [21–23]. Studies in *S*. *cerevisiae* were among the first to demonstrate a role for Hsp90 in client folding and it remains an ideal system to study Hsp90 and cochaperone function [16,17,24,25]. In *S*. *cerevisiae*, Hsp90 is encoded by two genes, *HSC82* and *HSP82*, and the two proteins are 97% identical. Expression of either one in an *hsc82hsp82* strain is sufficient for wild-type growth. We recently described a panel of mutations in the Hsc82 isoform that cause similar temperature-sensitive growth defects but varied effects on the ability to form distinct cochaperone complexes that are hallmarks of the Hsp90 folding cycle. The Hsc82 mutants also differed in their effect on activity on four clients: v-src kinase, heat shock transcription factor Hsf1, the DNA helicase Ssl2, and a protein involved in ribosome biogenesis, Utp21 [26]. In this study we used additional tests to determine whether mutants in each group share predictable phenotypes distinct from those in other groups. Over twenty years ago, Nathan and Lindquist described differing impacts of cochaperone overexpression on growth of yeast expressing *hsp82* mutations [27]. Our results suggest a simple explanation: there is a phenotypic shift in cochaperone requirements that correlates with whether Hsc82 mutants affect steps before or after formation of the closed, ATP-bound conformation. Sensitivity to the presence of NVP-AUY922 was also dependent on the conformational cycle: Hsc82 mutants that affect steps prior to formation of the closed, ATP bound conformation are hypersensitive to the drug, while mutants that affect post-closing steps are not. Our results provide critical in vivo evidence that cochaperones act as Hsp90 conformational pacemakers [17]. Further, we provide evidence that Hch1 and Cpr6 have opposing functions regulating formation of the closed state and clarify differences in Hch1 and Aha1 function. Our results demonstrate that the yeast system may be used to dissect how Hsp90 and cochaperones cooperate during the folding cycle, and may provide new insights into prior studies that show that overexpression or deletion of human cochaperones homologous to Hch1 and Cpr6 have opposing effects in models of neurodegenerative disease and cystic fibrosis [19,20].

## Results

We previously identified three groups of mutants in the Hsc82 isoform of Hsp90 that cause similar temperature sensitive growth defects but have differing effects on client activity and the ability of Hsc82 to form complexes associated with progression through the folding cycle [26]. The R46G and G309S alterations disrupt two separate contact sites between Hsp70 and Hsp90 in the loading complex when client is transferred from Hsp70 to Hsp90 [12]. Another mutation, K394E, also alters the contact site near G309 [28], but to simplify, only R46G and G309S are shown in most assays. The S481Y and A583T mutations disrupt the ability of Hsp90 to adopt the ATP-bound closed conformation characterized amino-terminal dimerization and Sba1 and Cpr6 interaction [21,26,29]. Hsp82-A587T, which mutates the residue of Hsp82 homologous to Hsc82-A583T **(S1 Table)**, was shown to be defective in nucleotide-induced amino-terminal dimerization [23]. We previously showed that other mutants in the vicinity of S481 and A587 also share similar phenotypes. One of those that is shown in some assays is T521I [21]. Three additional mutants, S25P, K102E and Q380K did not disrupt interaction with Hsp70, Sti1, Sba1 or Cpr6. Prior evidence suggests that they cause defects in regulation of ATP hydrolysis or reverting to the open, nucleotide-free conformation. S25 is adjacent to bound nucleotide [30], K102 is in the lid that closed over bound nucleotide, and Q380 is in a flexible loop that has been shown to regulate ATP hydrolysis [23,29,31]. For simplicity, we will refer to these three groups as loading, closing and reopening mutants. Here, we identify additional phenotypes that characterize each group of mutants.

### Effects of overexpression of *HCH1* correlates with conformation cycle

**Fig 1:**
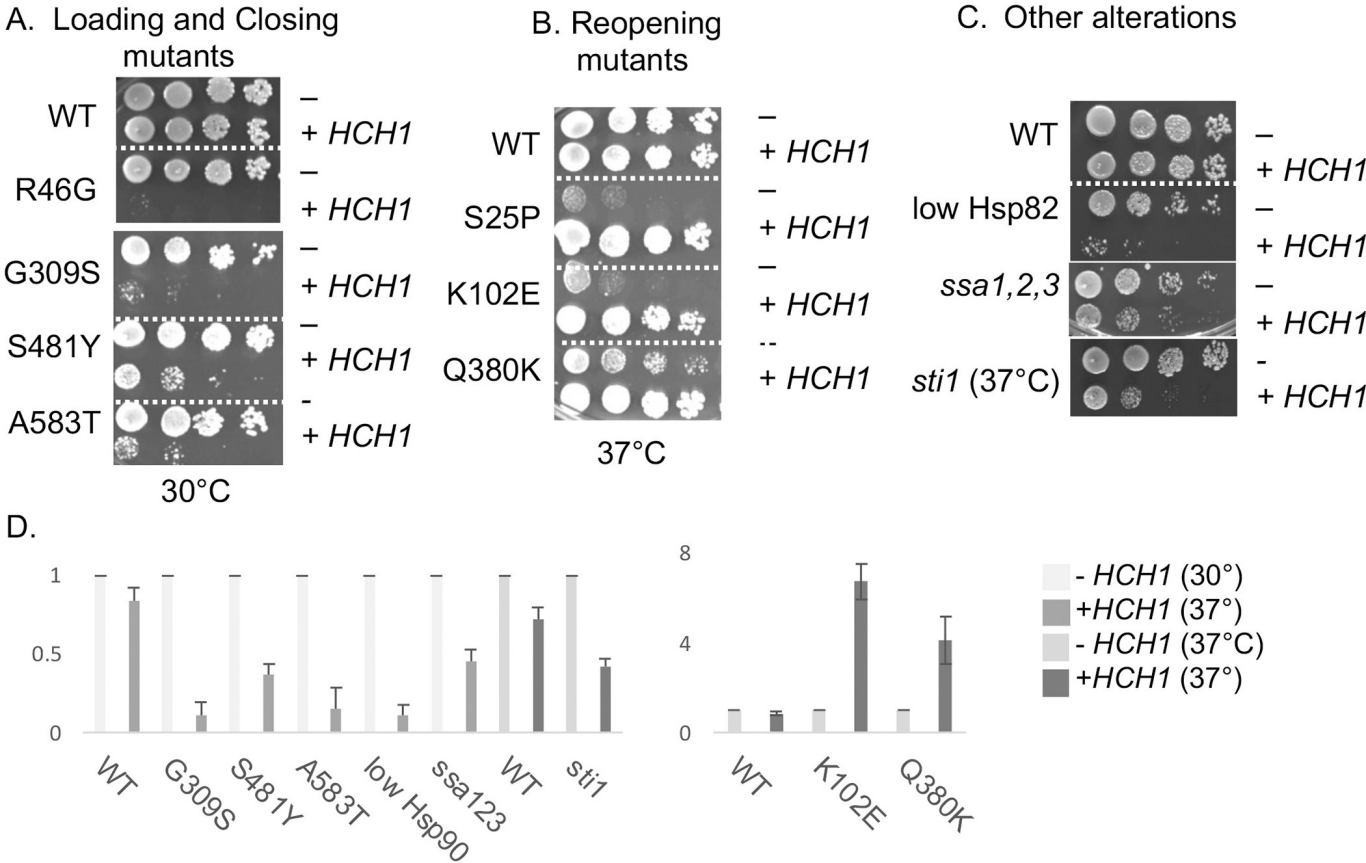
Effect of *HCH1* overexpression varies between mutant groups. Hsc82 mutants were expressed in strain JJ816 (*hsc82hsp82*). Cells expressing the indicated mutant were transformed with empty vector (p41KanTEF, -) or a plasmid overexpressing *HCH1* (p41KanTEF-Hch1-myc, + *HCH1*). Cells were grown overnight at 30°C, serially diluted 10-fold, plated on selective media (YPD + 200 μg/ml G418) and grown for two days at the indicated temperature. **A:** Mutants that alter residues required for direct interaction between Hsp70 and Hsp90 (R46G and G309S: loading mutants), or mutants that disrupt the ability of Hsc82 to interact with Sba1 and Cpr6 in the presence of AMP-PNP (S481Y, A583T: closing mutants). **B.** Mutants that alter residues associated with ATP hydrolysis or associated conformational changes (S25P, K102E, Q380K: reopening mutants). **C.** Additional strains expressing reduced levels of Hsp82, Hsp70 or containing a deletion in *STI1* (strains GRS4, JJ1480 and JJ623) were transformed with empty vector (p41KanTEF, -), a plasmid overexpressing *HCH1* (p41KanTEF-Hch1-myc, + *HCH1*), and grown as above. All strains were growth at 30°C except the strain lacking *STI1*. **D.** Effects of *HCH1* overexpression were quantified in three independent growth assays. Representative images of each sample set at shown. Left, mutations that affect loading and closing steps. Right, mutations that affect reopening steps.

*HCH1* was initially identified in a screen to identified genes that, when overexpressed, were able to rescue the growth defect of some, but not all *hsp82* alleles [27]. We overexpressed *HCH1* in strains expressing our set of *hsc82* alleles to determine if there was a correlation with our mutant groupings. Overexpression of *HCH1* resulted in a severe growth defect of mutants in the loading or closing group even at 30°C, the optimal growth temperature **(Fig 1A)**. In contrast, overexpression of *HCH1* rescued the temperature sensitive growth defects of the reopening mutants at 37°C **(Fig 1B)**. Thus, the shift in effect of *HCH1* overexpression correlates with transition into or out of the closed conformation. Our results are in complete agreement with the prior studies with *hsp82* alleles [27]. Importantly, the steady state level of Hsc82 was unaffected by *HCH1* overexpresssion **(S1 Fig).** Our hypothesis is that overexpression of *HCH1* antagonizes the loading and closing steps, exacerbating the defects of those mutants. In support of our hypothesis, we found that overexpression of *HCH1* also negatively affected growth of strains expressing reduced levels of *hsp82* [24], or the cytosolic Hsp70, Ssa *(ssa1ssa2ssa3)*, or deletion of *STI1*, which cooperates with Hsp70 during the loading phase **(Fig 1C)** [12,32]. The differences in yeast growth in the presence and absence of *HCH1* were quantified in a manner similar to prior studies and summarized in **Fig 1D** [33].

Since deletion of *HCH1* partially rescued the growth defect of cells expressing some *hsp82* alleles [34], we compared the growth of each *hsc82* mutant allele in isogenic *hsc82hsp82* and *hch1hsc82hsp82* strains. The growth defects of cells expressing mutants that disrupt the loading step (*hsc82*-R46G, G309S) or the closing step (S481Y, A583T, and T521I [26]) were strongly suppressed by *HCH1* deletion **(S2A Fig)**. In contrast, *hsc82-S25P* was inviable in the absence of *HCH1* **(S2B Fig)**, the growth of Q380K was reduced in the absence of *HCH1*, and the growth of K102E was only mildly affected by loss of *HCH1* **(S2C Fig)**. Overall, the effects of *HCH1* deletion were opposite of the effects of *HCH1* overexpression, as expected.

**Fig 2:**
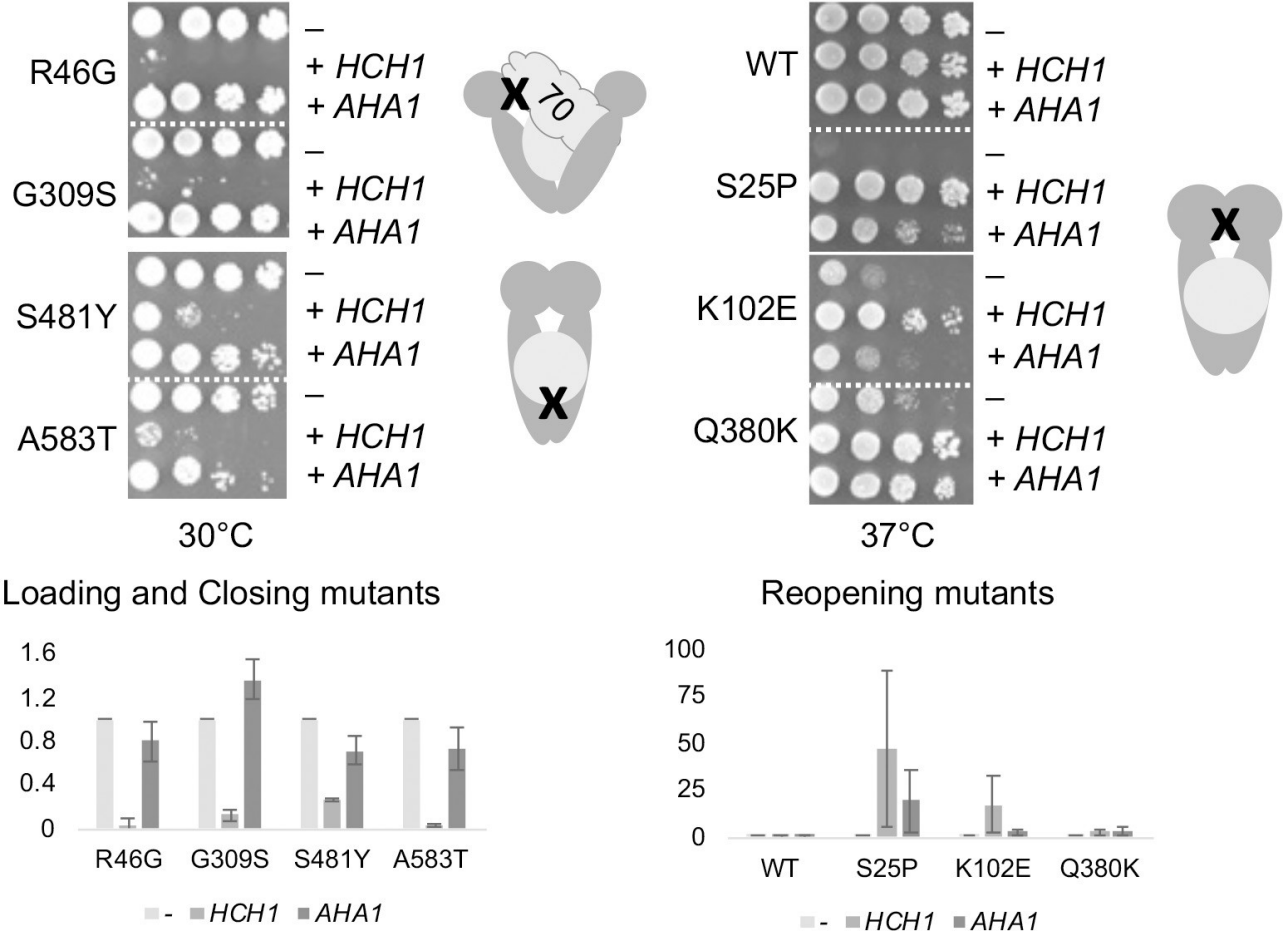
Effect of *AHA1* overexpression varies between mutant groups. As in Fig 1, except that cells were independently transformed with a plasmid overexpressing *AHA1* (p41KanTEF-Aha1-myc, +*AHA1*). Cells were then grown overnight at 30°C, serially diluted 10-fold, and plated on selective media (YPD + G418) and grown for two days at the indicated temperature. A cartoon shows the approximate location of the mutations within Hsp90. Dark gray, Hsp90 dimer. Circle, client, X, approximate site of mutations. 70, Hsp70. Below. Effects of *HCH1 of AHA1* overexpression were quantified using at least two independent growth assays.

Very little is known of the molecular mechanism behind Hch1 regulation of Hsp90. Hch1 can stimulate the ATPase activity of Hsp90, but weakly compared to the related cochaperone, Aha1. Hch1 is only present in some members of the subphylum, *Saccharomycotina*, while Aha1 is broadly present in eukaryotes. [35,36]. Aha1 possesses a C-terminal domain that contributes to its potent Hsp90 ATPase stimulation activity that has been well studied over the past 15 years [37]. Hch1 and the first domain of Aha1 interact with the middle domain of Hsp90 [35,38] and were once thought to be functionally homologous owing to their sequence and structural similarity, but functional differences have been identified [30,34,36]. As shown in **Fig 2**, overexpression of *AHA1* did not negatively affect the growth of the loading and closing mutants. Overexpression of *AHA1* had varied effects on the other mutants. The growth of S25P was partially rescued by overexpression of *AHA1*, as previously shown [30], but the effect of *HCH1* was stronger. Overexpression of *AHA1* had no effect on cells expressing *hsc82-K102E*. Finally, Hsc82-Q380K was fully rescued by either *AHA1* or *HCH1*.

**Fig 3:**
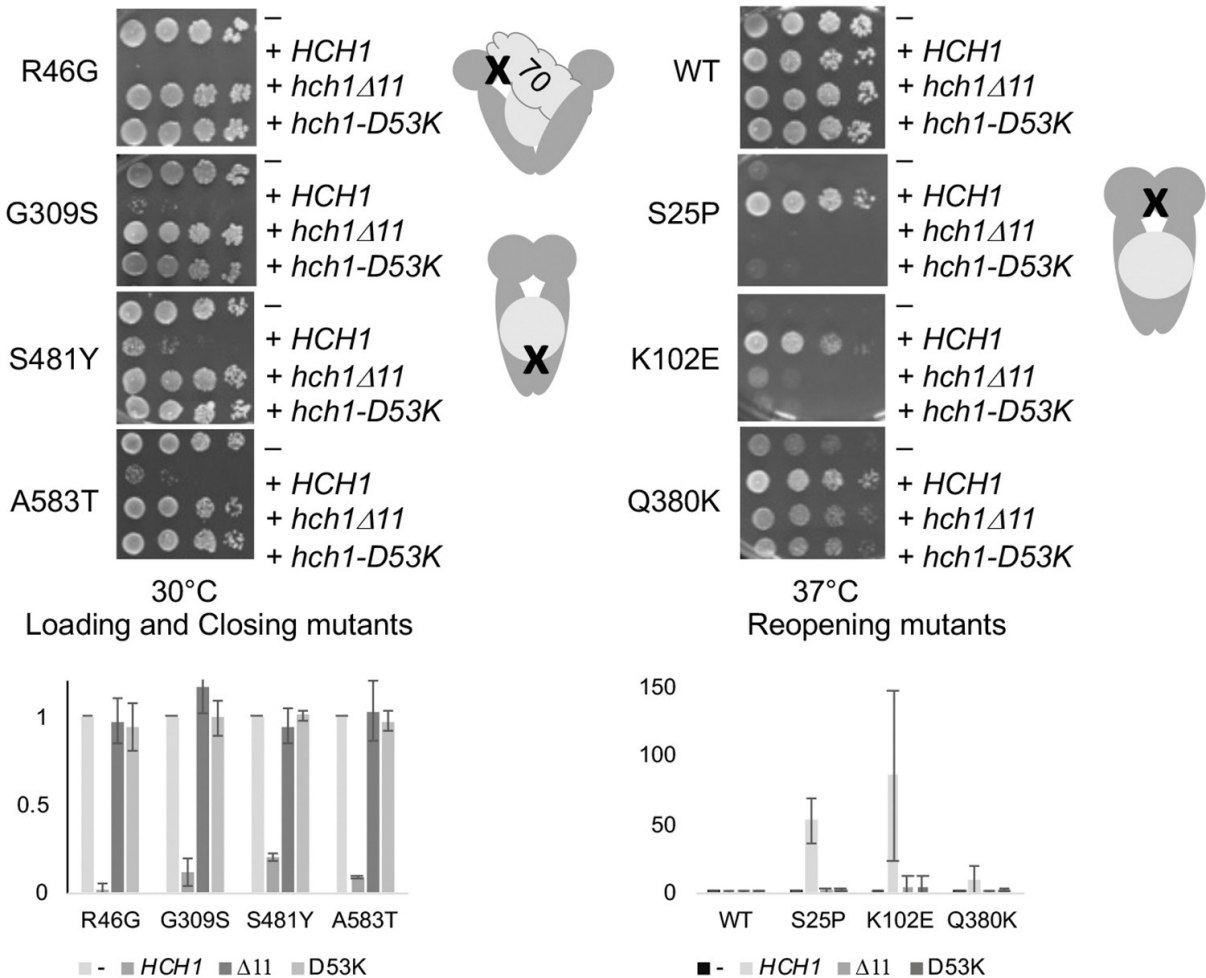
Deletion of the NxNNWHW sequence or the D53K alteration of Hch1 disrupts function. As in Fig 1, except that cells were also transformed with a plasmid overexpressing *HCH1* containing a deletion of the NxNNWHW sequence at the amino terminus (p41KanTEF-Hch1Δ11-myc, +*hch1Δ11*, or p41KanTEF-Hch1-D53K-myc, +*hch1-D53K*). Cells were grown overnight at 30°C, serially diluted 10-fold, and plated on selective media (YPD + G418) and grown for two days at the indicated temperature. Below. Effects of overexpression of wild-type or mutant Hch1 were quantified using at least two independent growth assays.

Aha1 and Hch1 have two conserved regions that are known to affect different aspects of function, (i.e. the RKxK and NxNNWHW motif). The conserved NxNNWHW sequence at the amino terminus of both Aha1 and Hch1 is required to fully stimulate the ATPase activity of Hsp90 [30]. Aha1 and Hch1 physically interact with the catalytic loop containing Hsp82-E381K and Hsc82-Q380K. The D53K charge reversal mutation near the RKxK motif impairs Aha1 and Hch1 binding to Hsp90 [39]. The D53K mutation in Hch1 or Aha1 had minimal effect on steady state levels **(S3 Fig),** while deletion of the NxNNWHW sequence in either Hch1 or Aha1 resulted in slightly reduced steady state levels. In all cases, deletion of the NxNNWHW or the D53K mutation in Hch1 eliminated the effect of *HCH1* overexpression **(Fig 3)**. Similarly, in those cases where *AHA1* overexpression had an effect (S25P and Q380K), deletion of the NxNNWHW sequence or the D53K mutation in *AHA1* eliminated the effects **(S4 Fig)**. Although deletion of NxNNWHW motif eliminated the effects of *HCH1* overexpression in vivo and also disrupted function in vitro [30], further studies are needed to establish the in vivo effects of higher expression of that mutant.

**Fig 4:**
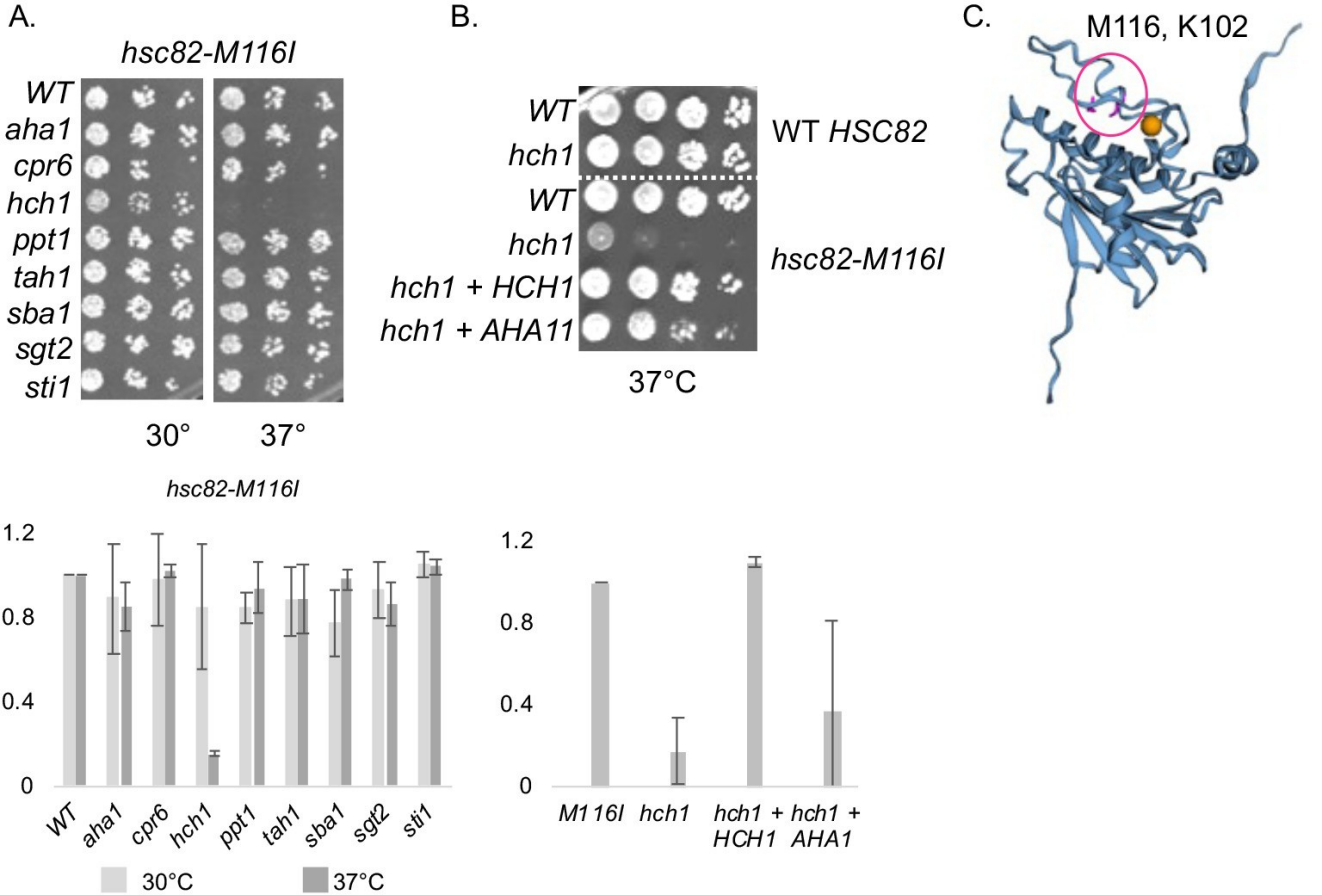
Identification of *hsc82-M116I*, which is specifically dependent on *HCH1* and alters a residue in the lid that closes over bound ATP. **A.** *hsc82-M116I* was expressed in isogenic *hsc82hsp82* strains that do not contain deletion of any cochaperones (WT) or contain individual deletion of the cochaperone listed. Strains are listed in S2 Table. Cells were serially diluted 10-fold and grown at the indicated temperature for two days. **B.** WT *HSC82* or *hsc82-M116I* were expressed in an *hsc82hsp82* strain (WT) or an *hch1hsc82hsp82* strain (*hch1*). Cells were transformed with empty vector (pRS426, -) or a plasmid overexpressing *HCH1* (pRS426-HCH1, + *HCH1*), grown overnight at 30°C, serially diluted 10-fold, plated on selective media and grown for two days at the indicated temperature. **C.** Location of M116 and K102 (shown in pick) mapped onto the closed AMP-PNP bound structure (PBD 2CG9). Figure generated with EZMOL [78], nucleotide shown in orange. Below. Growth assays were quantified using at least two independent growth assays.

Together, our results indicate that the effects of Hch1 and Aha1 are mediated through direct Hsc82 interaction, and that Hch1 has unique functions as well as some shared functions with Aha1. To gain additional insights into the function of Hch1, we used our existing mutant *hsc82* library to screen for mutants that exhibit temperature sensitive defects in an *hch1hsc82hsp82* strain. That resulted in identification of *hsc82*-M116I, which does not exhibit a 37°C growth defect unless *HCH1* is deleted. The growth defect is specific to *HCH1*, with no apparent defect in strains lacking any other nonessential cochaperones **(Fig 4A)**. The 37°C growth defect in the strain lacking *HCH1* is fully rescued by overexpressing *HCH1*, and partially rescued by *AHA1* overexpression (**Fig 4B)**. Similar to K102E this mutant is in the lid that closes over bound ATP (a.a. 94–125 in Hsc82) [29] **(Fig 4C)**, reinforcing our hypothesis that Hch1 plays a specific role in regulating access to the nucleotide binding pocket. Structural evidence shows that this residue is located on the other side of helix that makes contact with the NxNNWHW sequence [40], providing further evidence for a link between the NxNNWHW sequence and Hch1/Aha1 function.

### Additional analysis of cochaperone dependence

**Fig 5:**
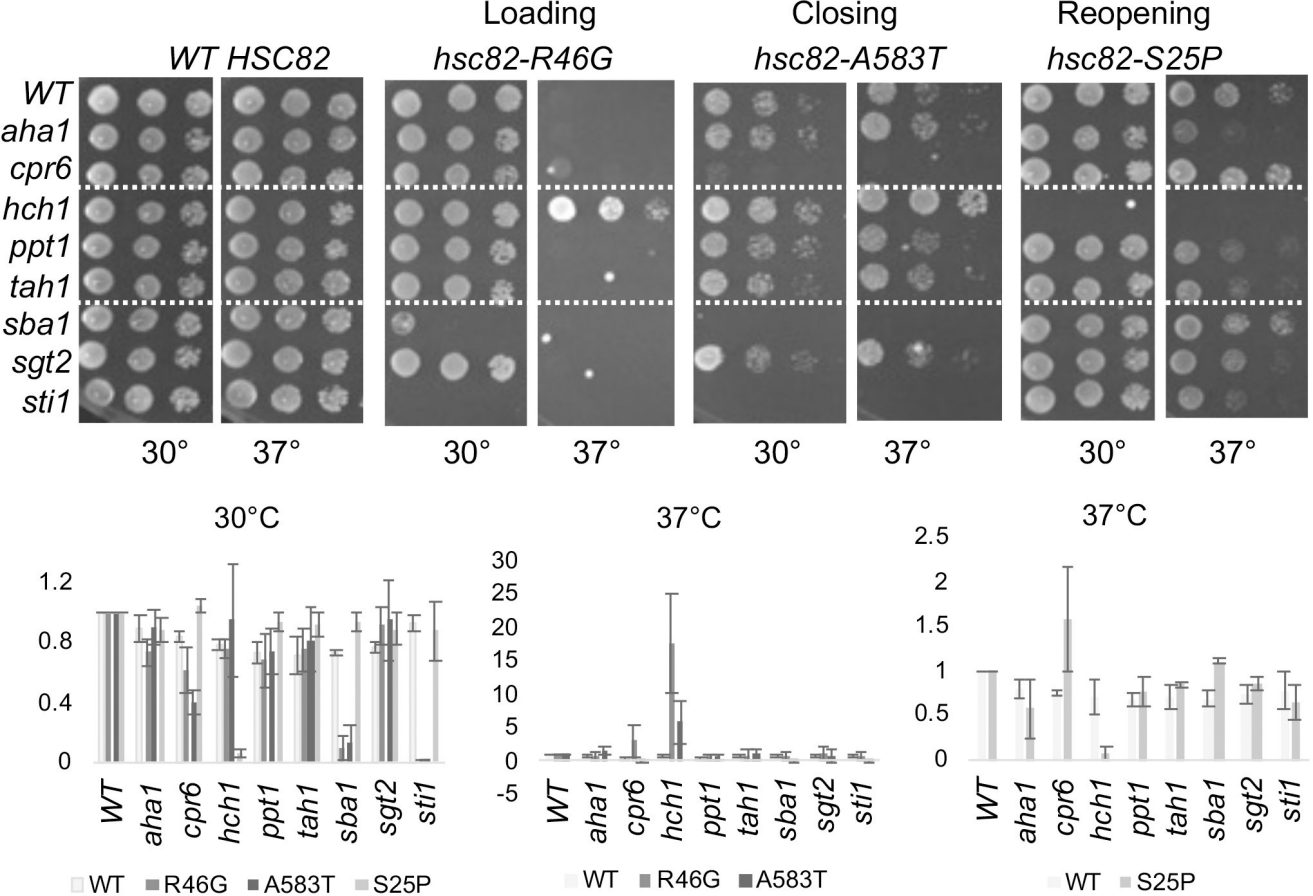
The effect of deletion of individual non-essential cochaperones varies according to mutant groupings. Plasmids expressing indicated WT or mutant forms of Hsc82 were expressed in isogenic *hsc82hsp82* strains that do not contain deletion of any cochaperones (WT) or contain individual deletion of the cochaperone listed. Cells were grown overnight at 30°C, serially diluted 10-fold, plated on rich media (YPD) and grown for two days at the indicated temperature. If no growth is shown on the 30° plate (such as with *hsc82-R46G* in the *sti1* strain), the cells were inviable. Below. Growth assays were quantified using at least two independent growth assays.

We next examined whether other cochaperones have critical roles regulating Hsc82 function in vivo. We transformed *hsc82* mutant alleles from each group into isogenic *hsc82hsp82* strains, each of which lacks one nonessential cochaperone: *AHA1*, *CPR6*, *HCH1*, *PPT1*, *TAH1*, *SBA1*, *SGT2* or *STI1* [16,17,41] **(Fig 5)**. Transformants were plated on 5-FOA, then subjected to serial dilutions to test growth. To ease comparison, when the combination was lethal (such as inviability of S25P in cells lacking *HCH1*), a blank spot was left in the serial dilutions. As expected, no growth defects are observed in any strains in the presence of WT *HSC82* [17]. As above, in cells expressing *hsc82-R46G*, loss of *HCH1* suppressed the growth defect at 37°C. Loss of *STI1* caused inviability and *SBA1* deletion caused a severe growth defect. Similar overall effects were observed upon expression A583T: deletion of *STI1* or *SBA1* caused inviability or severe growth defects, and deletion of *HCH1* partially suppressed the 37°C growth defect. Similar effects of cochaperone deletion on cells expressing *hsp82-A587T* were previously reported [42,43]. To determine if the growth patterns shown in **Fig 5** were consistent with our prior grouping of Hsc82 mutants, we compared all of the mutants within a group. As shown in **S5A Fig**, the loading mutants, R46G and G309S, show similar patterns. The K394E mutant is inviable in the absence of *STI1* [28], but the additional effects of loss of *SBA1* and *HCH1* are not observed at these temperatures. The closing mutants are similar to the loading mutants, but loss of *CPR6* has more dramatic effects in the closing mutants than the loading mutants **(S5B Fig)**. The strong dependence of the loading and closing mutants on *STI1*, *CPR6* and *SBA1* is consistent with the established functions: Sti1 is part of the loading complex and Cpr6 and Sba1 bind the closed complex [11,12,21].

The phenotypes of the reopening mutants were markedly different than those in the loading and closing groups. As shown above, *HCH1* was essential in cells expressing *hsc82-S25P*. *STI1* was not essential in the reopening mutants, although there were negative growth defects when this co-chaperone was deleted. Deletion of *SBA1* had little effect **(Fig 5)**. Growth defects of cells expressing all three mutants were suppressed by loss of *CPR6* **(S5C Fig)**. We examined two additional mutants with alterations in the same flexible loop as Q380K (L379S and E377A) [29,31]. All three residues, E377, L379 and Q380 are highly conserved **(S6A Fig)**. Hsc82-E377K, which contains an alteration homologous to *hsp82-E381K* [27], was inviable in our system [21], so we constructed E377A, which has a milder phenotype. L379S is a previously unreported mutant from the genetic screen that resulted in identification of R46G, and K102E other mutants [26]. All three mutations show temperature-sensitive growth defects that are enhanced in the absence of *HCH1* or are alleviated by *HCH1* overexpression, indicating they share characteristics of mutants in the reopening group **(S6B and S6C Fig)**. Also similar to Q380K, the E377A and L379S temperature sensitive growth defects are suppressed by loss of *CPR6* **(S5C Fig).**

### Opposing effects of *HCH1* and *CPR6*

**Fig 6:**
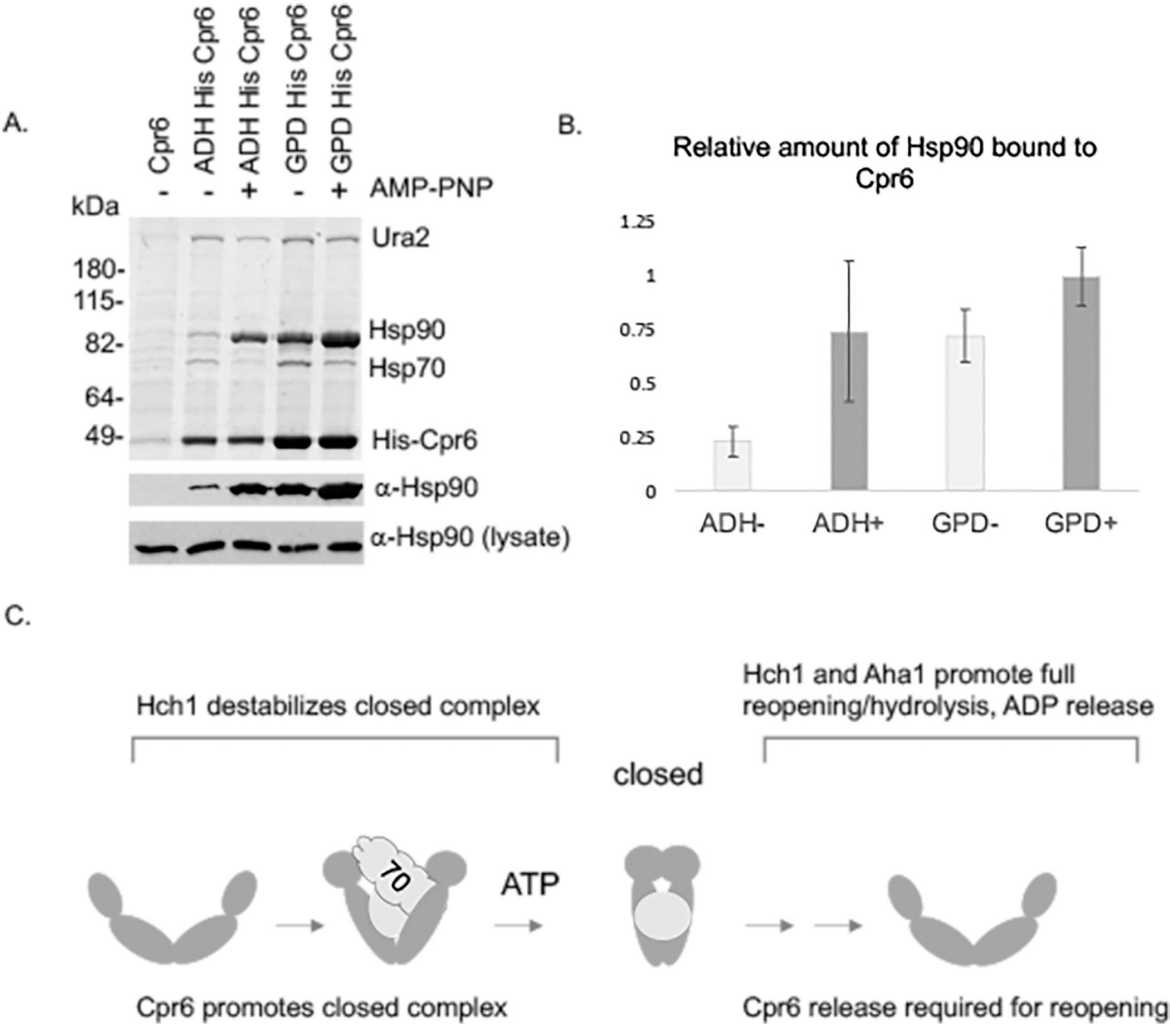
Overexpression of *CPR6* promotes Cpr6-Hsc82 interaction or stabilization in the absence of exogenous nucleotide. **A.** Strain JJ110 (*cpr6hsc82hs82*) expressed untagged Cpr6, His-*CPR6* under the ADH promoter or His-*CPR6* under the GPD promoter [42]. Cell lysates were incubated in the absence or presence of 5 mM AMP-PNP for 5 min. at 30°C prior to incubation with nickel resin. The stained gel is shown, as well as immunoblots showing Hsc82/Hsp82 bound to nickel resin or in whole cell lysates. **B**. Quantification of the relative levels of Hsc82/Hsp82 bound to His-Cpr6. **C**. Model for the opposing in vivo effects of Hch1 and Cpr6. Overexpression of *HCH1* exacerbates defects of the loading and closing mutants. Overexpression of *HCH1* or *AHA1* overcomes defects of the reopening mutants. Overexpression of *CPR6* promotes or stabilizes the closed conformation and release of Cpr6 is required for cycle progression.

The overall trends from the above suggest that Hch1 and Cpr6 have opposing functions in vivo. Our hypothesis is that Hch1 acts to destabilize the nucleotide-bound state. Thus, one possible opposing function of Cpr6 is to promote and/or stabilize the closed conformation. Crp6 is known to preferentially bind the carboxy-terminus of Hsc82 in the presence of AMP-PNP [21]. We previously analyzed the ability of Cpr6 containing a 6X-His tag (His-Cpr6) to interact with Hsc82/Hsp82 [42]. Isolation of His-Cpr6 complexes results in copurification of Hsp70, Hsp70, and the client Ura2. To examine the effect of *CPR6* overexpression, His-Cpr6 was expressed under the ADH promoter or the stronger GPD promoter. Both of these constructs overexpress Cpr6 relative to the endogenous promoter [42]. In the absence of exogenous nucleotide, the relative amount of Hsp90 bound to His-Cpr6 expressed under the GPD promoter is higher than when His-Cpr6 is expressed under the ADH promoter. Addition of AMP-PNP, a non-hydrolyzable form of ATP, results in an increase of Hsc82/Hsp82 interaction bound in both cases **(Fig 6A)**. The relative amounts of Hsc82/Hsc82 bound to His-Cpr6 in the presence or absence of AMP-PNP in triplicate experiments was quantified, with the results shown in **Fig 6B**. This supports our hypothesis is that overexpression of *CPR6* promotes and/or stabilizes the closed conformation. To provide additional evidence that enhanced Hsc82-Cpr6 interaction upon *CPR6* overexpression is functionally relevant, we transformed cells expressing *hsc82* mutants with plasmids expressing either ADH-*CPR6* or GPD-*CPR6*. Cells expressing WT *HSC82* or select mutants in the loading and closing group were unaffected by either level of *CPR6* overexpression. However, as shown in **S7 Fig**, transformation with GPD-*CPR6* (bottom), but not ADH-*CPR6* (top) resulted in a severe growth defect at 30°C in cells expressing S25P, Q380K or E377A, evidenced by very small colonies in the presence of GPD-*CPR6*. This indicates that *CPR6* overexpression exacerbates the growth defects of reopening mutants, the converse of how deletion of *CPR6* rescues the growth defects of these mutants **(S5C Fig)**.

Our model for the opposing in vivo effects of Hch1 and Cpr6 is shown in **Fig 6C**. Hch1 acts to destabilize the closed and/or nucleotide-bound state. In general, Hch1 and Aha1 promote reopening, although there may be some subtle functional differences during the reopening steps. Cpr6 promotes and/or stabilizes the closed conformation, and overexpression of Cpr6 may hinder reopening, possibly due to stabilized Hsc82 interaction. This hypothesis is consistent with our prior analysis of the effect of nucleotide of His-Hsc82 complexes. His-Hsc82 containing alterations in the catalytic loop, Q380A and N373A exhibited wild-type interaction with Cpr6 in the presence of AMP-PNP, but exhibited enhanced interaction with Cpr6 in the presence of ATP plus a regenerating system, suggesting a defect in Cpr6 release after hydrolysis [21].

Cpr6 has a paralog, Cpr7, which shares 38% percent identity. The two proteins have a similar domain structure and they bind Hsp90 with similar affinity, but they differ in some in vitro activities [44]. Unlike deletion of *CPR6*, deletion of *CPR7* results in slow growth, and growth defects of *cpr7* strains are not rescued by overexpression of *CPR6* [41,45]. We expressed loading (G309S), closing (A583T), or reopening (S25P, E377A, or Q380K) mutants in isogenic *hsc82hsp82*, *cpr6hsc82hsp82* or *cpr7hsc82hsp82* strains. In the loading or closing strains, deletion of either *CPR6* or *CPR7* resulted in strong growth defects, indicating that loss of either cochaperone exacerbates defects in obtaining the closed conformation **(S8 Fig)**. However, while loss of *CPR6* suppressed the 37°C growth defect of reopening mutants, a similar effect was not observed upon loss of *CPR7*. In addition, unlike overexpression of His-Cpr6, overexpression of His-Cpr7 does not result in significant Hsc82/Hsp82 interaction in the absence of AMP-PNP [42]. However, overexpression of either *CPR6* or *CPR7* caused a severe negative effect on the growth of *hsc82hsp82* cells expressing *hsc82-S25P* (**S8B Fig**). Together, these results suggest that there may be a similar requirement for Cpr6 and Cpr7 during progression through the folding cycle. However, the inability of overexpression of *CPR6* to rescue defects of *cpr7* cells and vice versa [41,42], suggests that they may function with distinct subpopulations of Hsp90 complexes or at distinct times during the cycle.

### Sensitivity to the Hsp90 inhibitor NVP-AUY922 correlates with the above grouping

**Fig 7:**
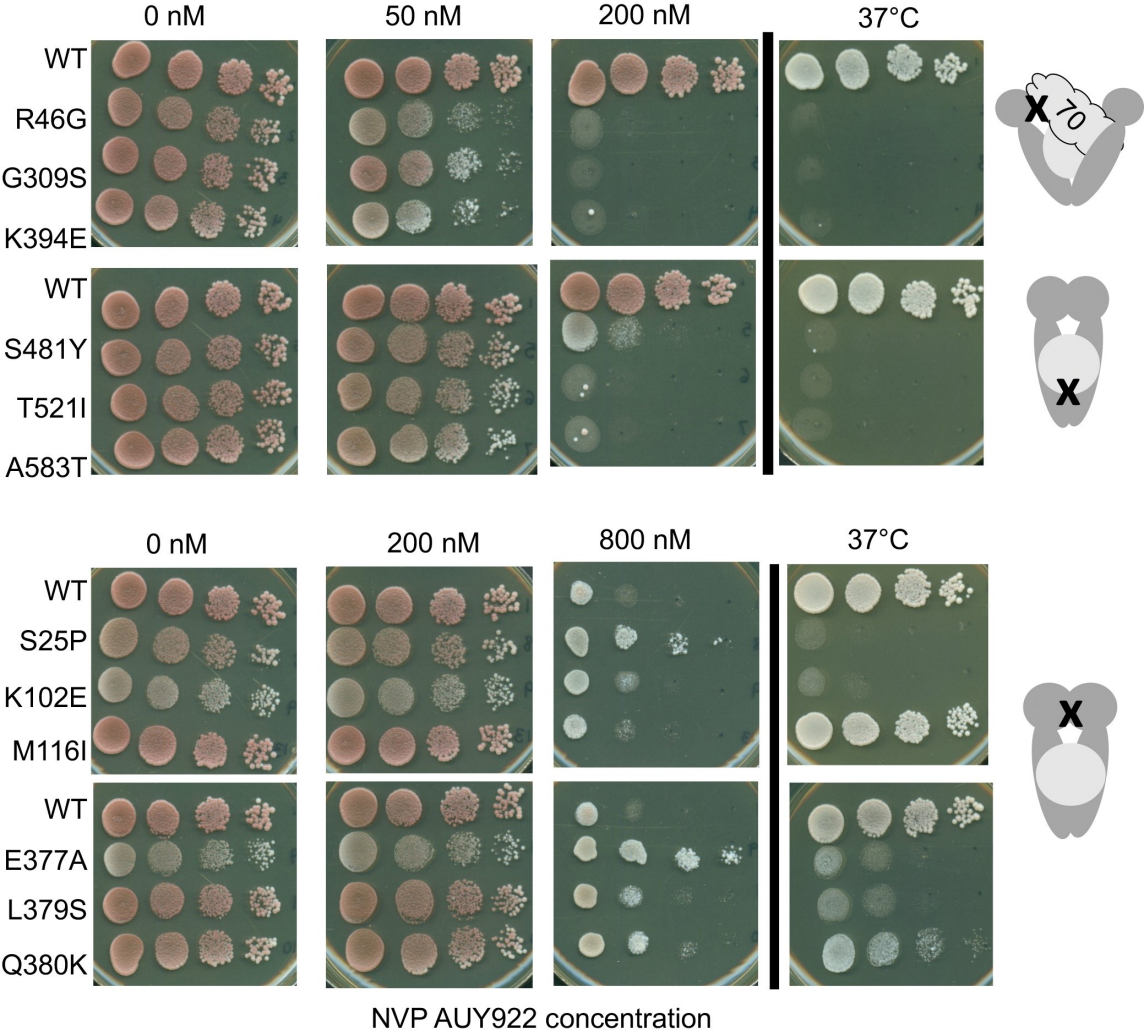
Sensitivity to Hsp90 inhibitor also correlates with mutant grouping. Strains were grown in YPD media and 10-fold serial dilutions were prepared and placed on agar plates with or without the indicated concentration of NVP-AUY922 where indicated and grown for 48 hours at the indicated temperatures. Growth assays were quantified using at least two independent growth assays (**S9 Fig**).

The results above reinforce our evidence that mutants within each group largely phenocopy each other. We next examined whether knowledge of how these mutants affect the conformational cycle could be used to examine the broader question of what dictates sensitivity to Hsp90 inhibitors that bind the nucleotide-binding pocket. This has been an important issue since the first report that tumor cells have increased affinity for Hsp90 inhibitors [46]. A prior report showed that overexpression of *HCH1* greatly increases sensitivity to the Hsp90 inhibitor NVP-AUY922 in yeast expressing wildtype Hsp82 [34]. Since we observed strong *HCH1*-dependent effects on growth, we examined whether cells expressing *hsc82* mutants exhibit varied drug sensitivity **(Figs 7 and S9)**. The loading mutants (R46G, G309S and K394E) were sensitive to the drug, showing reduced growth in the presence of 50 nM NVP-AUY922 and inviability at a higher concentration (200 nM). The closing mutants (S481Y, T521I and A583T) showed only mild inhibition in the presence of 50 nM but, unlike cells expressing WT Hsc82, were inviable at the higher concentration. In contrast, the post-closing mutants were highly resistant to the effects of the inhibitor, showing little or no growth defects in the presence of 200 nM concentration of the drug. Indeed, at very high concentration (800 nM), S25P, E377A and Q380K may confer enhanced resistance to the drug relative to wild-type cells, but further tests are required to confirm this result. Thus, Hsp90 in the open conformation is more susceptible to this type of Hsp90 inhibitor, while Hsp90 in the closed conformation is relatively resistant to the effects of the drug.

## Discussion

We developed a robust system to study Hsp90 and cochaperone interactions in in the genetically tractable *S*. *cerevisiae* and provide genetic evidence for the model of Hsp90 and cochaperone action during the folding cycle. Hsp90 mutations that target distinct steps in the pathway have phenotypically distinguishable phenotypes that include client specificity, cochaperone dependence and sensitivity to an Hsp90 inhibitor. Client is loaded onto Hsp90 in a process facilitated by Hop/Sti1. In the presence of ATP, Hsp70 and Hop/Sti1 dissociate, and Hsp90 forms the closed conformation characterized by p23/Sba1 and Cpr6 interaction. Hsp90 mutants that disrupt the loading or closing steps exhibit growth defects in the absence of cochaperones that are critical components of these steps [11,12,21,23,28]. The loading and closing mutants or cells with reduced levels of Hsp90 or Hsp70 or deletion of *STI1* were similarly negatively affected by *HCH1* overexpression, suggesting Hch1 negatively regulates progression through the early steps of the cycle. The reopening mutants were initially grouped primarily due to their locations in regions critical for ATP hydrolysis and ADP release. K102 is located in the lid that cover the ATP binding pocket [29]. The loop that contains Hsc82-Q380 comes in contact with the ATP binding pocket to help promote hydrolysis [14,29,47,48]. S25 and Q380 make intermolecular contacts with one another at the interface between the two Hsp90 N domains [40]. Evidence presented here supports and reinforces the overall grouping: all mutants within this group are rescued by *HCH1* overexpression or *CPR6* deletion. Overexpression of *AHA1* also rescued some of the reopening mutants. Our demonstration of how cochaperones enhance or alleviate defects of Hsc82 mutants in vivo are consistent with in vitro studies that describe cochaperones as "conformational pacemakers” critical for the correct timing of the folding cycle [14,17,49–51].

Our hypothesis is that Hch1-mediated destabilization of the nucleotide bound state negatively regulates progression through the folding cycle and may also enhance exchange at the end of the cycle. Kinetic analysis of ATPase stimulation by Hch1 or Aha1 found that Hch1, but not Aha1, increased the apparent Km for ATP [30]. While the NxNNWHW sequence in Hch1 is important for regulating nucleotide affinity, its absence did not influence the rate of lid closure, and we speculated that it may play a role in ADP release [30]. Analysis of available structures show that there are multiple lid conformations [52], and it is possible that the assay we used did not capture the appropriate change in lid movement. The K102E and M116I mutations alter residues in the lid and are highly dependent on *HCH1*, consistent with this role. Two Hsp82 mutants, A41V, which bound AMP-PNP much less tightly than wild-type Hsp82, and T101I, which is predicted to disfavor dimerization, were also negatively affected by *HCH1* overexpression [23,27], providing support for our hypothesis that Hch1 has a role in nucleotide interaction and cycle progression. Similarly, *HCH1* rescued growth defects of cells expressing *hsp82-T22I and hsp82-E381K* [27]. Hsp82-E381K alters the residue homologous to Hsc82-E377A, which is also rescued by *HCH1* overexpression. Hsp82-T22I has been shown to favor the closed conformation [23]. A more general role for Hch1 that affects interaction with both ATP and ADP may explain the observed increased sensitivity of yeast cells overexpressing *HCH1* to Hsp90 inhibitors, but it is possible that the different inhibitors have differing abilities to bind subtly distinct closed conformations of the various Hsp90 mutants. [34]. Similar to our results, Piper et al found that cells expressing *hsp82-A583T* were more sensitive, while cells expressing *hsp82-E381K* were fairly resistant to both geldanamycin but not radicicol, similar to our results with NYP-AUY922, but cells expressing *hsp82-T22I* were sensitive to geldanamycin but not radicicol [53]. Piper et al also found that deletion of *PDR5*, a plasma membrane ATP binding cassette transporter increased sensitivity to RD and GA. Additional studies are needed to determine whether deletion of *PDR5* changes the overall pattern of *hsc82* mutant sensitivity to NYP-AUY922. The paper by Piper et al, also showed that deletion of *STI1* or *CPR7*, combined with deletion of either *HSC82* or *HSP82* had some effects on drug sensitivity [53]. Additional studies are also needed to determine how cochaperone deletion or overexpression affects sensitivity of to NYP-AUY922. Further studies are also needed to determine how specific Hsp90 isoforms are affected by cochaperone deletion or overexpression in the context of Hsp90 inhibitor sensitivity.

In our model **(Fig 6C)**, the loading and closing mutants are delayed in progressing to the closed step, increasing susceptibility to ATP release. The loading and closing mutants are rescued by *HCH1* deletion because once ATP binds, it is less likely to release, helping to drive cycle progression. Deletion of genes encoding other proteins known to act early in the folding cycle also results in increased sensitivity to Hsp90 inhibitors [53,54]. This includes *STI1* and *SSE1*, a nucleotide exchange factor for the Hsp70 Ssa. Interestingly, deletion of yeast *AHA1* does not affect sensitivity to inhibitors, but silencing of mammalian AHA did increase sensitivity to an Hsp90 inhibitor [55], suggesting that yeast Hch1 and mammalian Aha1 may have a similar role modulating nucleotide affinity. Consistent with our hypothesis, another study showed that Aha1 induces intermediate conformational rearrangements around the ATP-binding pocket of Hsp90 that primes the domain for nucleotide trapping [56]. It is possible that Hch1 is needed in yeast to compensate for altered conformational dynamics that exist between yeast Hsc82 and human Hsp90, or species-specific differences in ATPase rates, which is fastest in yeast compared to human Hsp90 and E. coli HtpG [22,23].

There are still may questions regarding Aha1 and Hch1 functions. Aha1 has also been shown to function early in the cycle, cooperating with Cpr6 to displace Sti1 in order to assist progression of the folding cycle, and promoting conformational changes in Hsp90 [50,57]. Our hypothesis is that Hch1 and Aha1 may both enhance the acquisition of the N terminally bound state through repositioning of the catalytic loop [30,34]. Both of the conserved sequence motifs (i.e. the RKxK and NxNNWHW motif) are oriented towards the N terminal domains of the Hsp90 dimer [39]. In a recent structure, the Aha1 C terminal domain was observed to interact with both middle domains of the dimer. Interestingly, the Aha1 C terminal domain has a Bet v1 fold that is characterized by a hydrophobic binding pocket [40,58]. Upon nucleotide binding, Hsp90 adopts a symmetrical, N terminally dimerized state and both domains of Aha1 undergo a rearrangement, altering contacts with residues near the nucleotide binding pocket and the catalytic loop and repositioning of the interaction with the hydrophobic pocket [14,29,47,48]. This is noteworthy because the NxNNWHW motif of Aha1, and presumably that of Hch1, is in direct contact with K102 in the nucleotide-bound state. Q380 is in the catalytic loop that is reoriented towards the N domain of the opposite protomer upon nucleotide, client, and/or Aha1 binding [11,12,29,31,39,40]. This places it in very close proximity to the S25 residue in the opposite protomer. The phenotypes associated with S25 and Q380 are suppressed by both Hch1 and Aha1, suggesting that both cochaperones may regulate the position of the catalytic loop and/or communication with the opposite N domain. In contrast, suppression of the phenotypes associated with K102E, in the lid, are only affected by Hch1. This may suggest that Hch1 may play a specific role in regulating access to the nucleotide binding pocket in a way that Aha1 does not. Future studies are needed, but these mutants may be valuable tools to elucidate the timing of the reopening events, including ATP hydrolysis in each monomer, lid opening, ADP release and client release.

In general, our results suggest that a significant number of the Hsc82/Hsp82 temperature sensitive mutants that have been described have defects related to conformational changes that may be at least partially rescued by alterations in levels of certain cochaperones [21,25, 59]. With regard to effects of cochaperone overexpression, alteration of homologous residues in Hsc82 and Hsp82 have similar effects [27,28,34,36]. However, other differences between the two isoforms, including client specificity, have been identified [60], and further analysis is needed to determine whether the ATPase activity of similar mutants in the two isoforms have similar effects. Importantly, not all available mutants fit within these three established groups. This includes, Hsc82-W296A/Hsp82-W300A and Hsc82-G424D [26,61]. Other mutations in Hsp82 have been identified but have not yet been characterized with regard to effects of cochaperone overexpression [62–64].

Our results indicate that the effect of Hsp90 mutation on the conformational cycle may be a better indicator of the overall effect on function than changes in inherent ATPase activity. This is consistent with prior reports that ATPase activity is not a good indicator of client selectivity [62]. Prior studies also found no correlation between ATPase activity and ability to be rescued by *HCH1* overexpression [23,27,31]. It has been hypothesized that a post-translational modification of a residue in the carboxy terminus of human Hsp90 that affects formation of the closed state eliminated the need for Hch1 [65]. It is well known that post-translational modifications may regulate conformational changes [64,66–68]. Hsp90 interaction with cochaperones affects affinity of Hsp90 interaction with inhibitors [46], and the conformation and post-translational modifications of Hsp90 and the growth state of cells also contributes to sensitivity to inhibitors [69–72]. Broad inhibitors of Hsp90 function, such as those that target ATP-binding pocket, have anti-cancer activity, but are not widely used [6,8–10]. However, Pimitespib, an alternate Hsp90 inhibitor that binds the ATP-binding pocket, has recently been shown to meet its primary endpoint in a randomized Phase III clinical trial, which represents an important milestone in the development of Hsp90 inhibitors [73]. Pimitespib has recently been approved for treatment of Gastrointestinal Stromal Tumor (GIST) in Japan. Alternative approaches to selective inhibition of Hsp90 have been proposed, such as blocking interaction with select cochaperones, targeting other sites on Hsp90, or isoform-specific inhibitors [74–76]. Our results suggest that the combination of a compound that slows cycle progression, such as one targeting Hsp70-Hsp90 interaction, plus an inhibitor that targets the ATP-binding pocket might be very effective therapeutic strategy for Hsp90 inhibition.

Hsp90 is critical for folding and regulation of proteins involved in diseases like cancer, Alzheimer’s disease, and cystic fibrosis [5–7]. Overall, our results suggest that opposing effects of silencing or overexpression of Aha1 and Cpr6 homologs in in mammalian cells may be linked to differing effects on Hsp90 conformation [19,20]. In mammalian cells, knockdown of *AHA1* was associated with increased levels of CFTR-F508delta [20]. A prior study showed that GR activity in yeast decreased upon deletion of Sti1, Sba1 or Cpr6, and increased upon deletion of *HCH1* or *AHA1* [17]. In addition, a prior study showed that deletion of *HCH1* partially suppressed the growth defects of *cpr7* cells [16], which is similar to our results that suggest opposing roles of Cpr6 and Hch1. Additional studies are needed to understand how cochaperones cooperate with Hsp90 regulate client function.

## Materials and methods

### Media, chemicals, antibodies, plasmids and yeast methods

Yeast were transformed by lithium acetate methods and were grown in either YPD (1% Bacto yeast extract, 2% peptone, and 2% dextrose) or defined synthetic complete media supplemented with 2% dextrose. Growth was examined by spotting 10-fold serial dilutions of yeast cultures on appropriate media, followed by incubation for two days at 30°C or 37°C. 5-fluorootic acid (5-FOA) was obtained from Toronto Research Chemicals. G418 was obtained from Sigma. Plasmids expressing non-His-tagged or His-tagged Hsc82 have been described, as have plasmids expressing His-tagged Cpr6 or Cpr7 [26,42]. The *hsc82-M116I* mutation was isolated using the *hsc82* mutant library described previously [26]. Plasmids p41KanTEF-Hch1-myc and p41KanTEF-Aha1-myc have been described [30]. The D53K mutation was introduced into Hch1 and Aha1 using site-directed mutagenesis and the coding sequence was sequenced completely to confirm the mutation. Additional plasmids overexpress genomic fragments of *HCH1* or *AHA1* in the multicopy plasmid pRS426 [77]. Studies with the Hsp90 inhibitor NVP-AUY922 (LC labs) were performed as described [34]. All strains are isogeneic to W303 and are listed in **S2 Table**. In general, the plasmid containing desired wildtype or mutant Hsc82 was transformed into JJ816 (or other strain as indicated), grown for two days, then struck out onto 5-FOA to counterselect for the YEp24-*HSP82* plasmid prior to testing growth phenotypes. Analysis of the growth defects were quantified using a technique similar to one previously described [33]. With the exception of the growth assays shown in **S5 Fig**, all growth tests were conducted at least twice, with representative examples shown. Growth was normalized to that of cells expressing the indicated variant of *HSC82* in the otherwise wild-type *hsc82hsp82* strain background or in the presence of empty vector.

### Isolation of His-Cpr6 complexes and immunoblot analysis

His-Cpr6 complexes were isolated as described [42]. Briefly, yeast empty vector (p416GPD) or expressing WT or mutant His-Cpr6 under the ADH or GPD promoters were grown overnight to an OD_600_ of 1.2–2.0. Cells were harvested, washed with water and resuspended in lysis buffer (20 mM Tris, pH 7.5, 100 mM KCl, 5 mM MgCl_2_ containing a protease inhibitor mixture (Roche Applied Science)). Cells were disrupted in the presence of glass beads with 8 x 30 sec pulses. Complexes were isolated by incubation with nickel resin (1 hour with rocking, 4°C) followed by washes with lysis buffer plus 0.1% Tween-20 and 35 mM imidazole. Nickel resin was boiled in SDS-PAGE sample buffer and protein complexes were separated by gel electrophoresis followed by Coomassie Blue staining and/or immunoblot analysis using indicated antibodies. Coomassie Blue stained gel was converted to grayscale in Fig 6A. The assay shown in Fig 6A to study His-Cpr6 interaction with Hsc82/Hsp82 in the presence or absence of AMP-PNP was conducted with three independent sets of samples and the relative intensities of the bands representing His-Cpr6 and Hsp90 in the Coomassie Blue stained gel was determined using ImageJ. An additional set of triplicate samples that compared the levels of Hsc8/Hsp82 interaction with His-Cpr6 expressed in the absence of AMP-PNP was also used in the analysis. For additional immunoblot analysis, cells (0.5 O.D._600_ units) were resuspended in phosphate-buffered saline containing 1 mM phenylmethylsulfonylfluoride and disrupted with glass beads in the presence of SDS and triton X-100.Chemiluminescence immunoblots were performed according to the manufacture’s suggestions (Pierce, Rockford, IL). The polyclonal antisera against Hsc82/Hsp82 has been described [26]. Additional monoclonal antibodies were obtained from Invitrogen (anti-Pgk1 459250 and anti-Myc MA1-21316).

## Supporting information

S1 Table Comparison of Hsc82 and Hsp82 isoforms. Accession numbers used: *S*. *cerevisiae* Hsc82 KZV09036.1; *S*. *cerevisiae* Hsp82 NP_015084.1; *Homo sapiens* Hsp90 alpha (AAI21063.1).
(DOCX)

S2 Table Strains used in this study. All are isogenic to W303.
(DOCX)

S1 Fig *HCH1* overexpression does not affect steady state levels of Hsc82 mutants. Extracts from cells expressing WT *HSC82* or indicated *hsc82* mutant were separated by SDS-PAGE and immunoblotted with a polyclonal antisera specific for Hsc82/Hsp82. R46G and G309S were not included due to the severe growth defect in the presence of *HCH1*. An antibody against Pgk1 was used as a loading control (Invitrogen catalog #459250).
(TIF)

S2 Fig Effect of *HCH1* deletion is opposite of the effect of *HCH1* overexpression. His-Hsc82 mutants were transformed into isogenic *hsc82hsp82*/YEp24-*HSP82* (JJ816) or *hch1hsc82hsp82*/YEp24-*HSP82* (JJ111) strains. **A** and **C**. Transformants were grown in the presence of 5-FOA to cure the YEp24-*HSP82* plasmid, grown overnight at 30°C, serially diluted 10-fold, and plated on YPD and grown for two days at the indicated temperature. Right. Quantification of replicate growth assays. **B.** Transformants expressing wild-type *HSC82* or *hsc82-S25P* were grown in the presence of 5-FOA for 3 days.
(TIF)

S3 Fig Expression of WT and mutant forms of Hch1 and Aha1. Plasmids p41KanTEF-Hch1-myc and p41KanTEF-Aha1-myc, or the empty vector (p41KanTEF) were transformed into the wildtype strain JJ762. Cell extracts were separated by SDS-PAGE and immunoblotted with a monoclonal antibody specific for Myc (Invitrogen MAI-21316). An antibody against Pgk1 (Invitrogen 459250) was used as a loading control.
(TIF)

S4 Fig Effect of *AHA1* mutation on ability to rescue growth of *hsc82* mutants. As in Fig 2, except that cells were also transformed with a plasmid overexpressing WT *AHA1* (p41KanTEF-Aha1-myc, +*AHA1*) or plasmids that delete the NxNNWHW of Aha1(*aha1-Δ11*) or containing the D53K alteration (*aha1-D53K*). Cells were then grown overnight at 30°C, serially diluted 10-fold, and plated on selective media (YPD + G418) and grown for two days at the indicated temperature. Right. Quantification of replicate growth assays
(TIF)

S5 Fig The effect of deletion of individual non-essential cochaperones is similar within mutant groups. As in Fig 5, Plasmids expressing indicated WT or mutant forms of Hsc82 were expressed in isogenic hsc82hsp82 strains that do not contain deletion of any cochaperones (WT) or contain individual deletion of the cochaperone listed. Left. Cells were grown overnight at 30°C, serially diluted 10-fold, plated on rich media (YPD) and grown for two days at the indicated temperature. If no growth is shown on the 30° plate (such as with hsc82-R46G in the sti1 strain), the cells were inviable. A. Loading mutants. B. Closing mutants. C. Reopening mutants. Right. Quantification of growth assays.
(TIF)

S6 Fig Mutations in the conserved loop have similar phenotypes. **A.** Conservation of residues in catalytic loop. Accession numbers used: *S*.*cerevisiae* Hsc82 KZV09036.1; *S*. *cerevisiae* Hsp82 NP_015084.1; *Homo sapiens* Hsp90 alpha (AAI21063.1); *Homo sapiens* Hsp90 beta (NP_031381.2); *E*. *coli* HtpG (NP_415006.1). **B.** His-Hsc82 mutants were transformed into isogenic *hsc82hsp82*/YEp24-*HSP82* (JJ816) or *hch1hsc82hsp82*/YEp24-*HSP82* (JJ111) strains. Transformants were grown in the presence of 5-FOA to cure the YEp24-*HSP82* plasmid, grown overnight at 30°C, serially diluted 10-fold, and plated on YPD and grown for two days at the indicated temperature. **C.** Cells expressing the indicated mutant were transformed with empty vector (pRS426, -) or a plasmid overexpressing *HCH1* (pRS426-HCH1, + *HCH1*). Cells were grown overnight at 30°C, serially diluted 10-fold, plated on selective media and grown for two days at the indicated temperature. Right. Quantification of replicate growth assays
(TIF)

S7 Fig Overexpression of *CPR6* causes severe growth defects in cells expressing mutants in the reopening group. Hsc82 mutants were expressed in strain JJ816 (*hsc82hsp82*). Strains were transformed with plasmids pRS416ADH-His-*CPR6* (top) or pRS416GPD-His-*CPR6* (bottom). Transformants were grown for up to four days at 30°. Extremely small colonies are visible in cells expressing the combination of reopening mutants and GPD-His-*CPR6*.
(TIF)

S8 Fig Comparison of the effects of *CPR6* and *CPR7*. **A.** Hsc82 mutants were expressed in strain JJ816 (*hsc82hsp82*), JJ110 (*cpr6hs82hsp82*) or JJ149 (*cpr7hsc82hsp82*). Transformants were grown in the presence of 5-FOA to cure the YEp24-*HSP82* plasmid, grown overnight at 30°C, serially diluted 10-fold, and plated on YPD and grown for two days at the indicated temperature. **B**. Hsc82 mutants expressed in strain JJ816 (*hsc82hsp82*), were transformed with empty vector, pRS416ADH-His-*CPR6* or pRS416GPD-His-*CPR7*. Cells were serially diluted 10-fold, and plated on selective media and grown for two days at 30°C. Below. Quantification of replicate growth assays
(TIF)

S9 Fig Quantification of replicate growth assays in Fig 7. Strains were grown in YPD media and 10-fold serial dilutions were prepared and placed on agar plates with or without the indicated concentration of NVP-AUY922 where indicated and grown for 48 hours at the indicated temperatures.
(TIF)

S1 Data Excel file containing quantification of growth assays. ImageJ was used to compare growth of different strains or quantify protein levels as described in Materials and Methods.
(XLSX)
