# Correction: Hsp90 mutants with distinct defects provide novel insights into cochaperone regulation of the folding cycle

**DOI:** 10.1371/journal.pgen.1011850

**Published:** 2025-09-04

**Authors:** Rebecca Mercier, Danielle Yama, Paul LaPointe, Jill L. Johnson

In the Sensitivity to the Hsp90 inhibitor NVP-AUY922 correlates with the above grouping subsection of the Results, there are errors in the sixth, seventh, eighth and ninth sentences of the first paragraph. The correct sentences are: The loading mutants (R46G, G309S and K394E) were sensitive to the drug, showing reduced growth in the presence of 50 µM NVP-AUY922 and inviability at a higher concentration (200 µM). The closing mutants (S481Y, T521I and A583T) showed only mild inhibition in the presence of 50 µM but, unlike cells expressing WT Hsc82, were inviable at the higher concentration. In contrast, the post-closing mutants were highly resistant to the effects of the inhibitor, showing little or no growth defects in the presence of 200 µM concentration of the drug. Indeed, at very high concentration (800 µM), S25P, E377A and Q380K may confer enhanced resistance to the drug relative to wild-type cells, but further tests are required to confirm this result.

In Fig 7, the 0 nM, 200 nM, and 800 nm values are incorrect it should be 0 µM, 50 µM and 800 µM. Please see the correct Fig 7 here.

**Fig 7:**
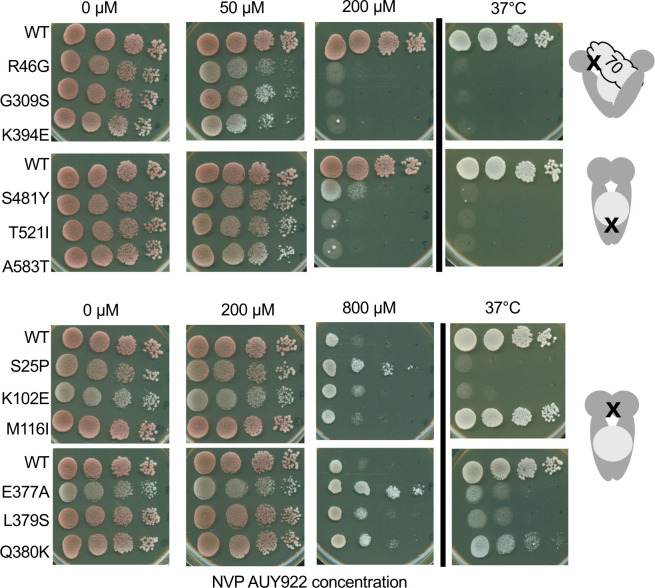
Sensitivity to Hsp90 inhibitor also correlates with mutant grouping. Strains were grown in YPD media and 10-fold serial dilutions were prepared and placed on agar plates with or without the indicated concentration of NVP-AUY922 where indicated and grown for 48 hours at the indicated temperatures. Growth assays were quantified using at least two independent growth assays (Fig S9).
