# Supplementary material for: Hsp90 mutants with distinct defects provide novel insights into cochaperone regulation of the folding cycle
Source: PLoS Genet. 2023 May 25;19(5):e1010772. doi: 10.1371/journal.pgen.1010772 (PMC10246838; doi:10.1371/journal.pgen.1010772)
Supplement: S1 Table — Accession numbers used: S. cerevisiae Hsc82 KZV09036.1; S. cerevisiae Hsp82 NP_015084.1; Homo sapiens Hsp90 alpha (AAI21063.1). (DOCX) [file pgen.1010772.s001.docx]

**S1 Table. Comparison of Hsc82 and Hsp82 isoforms**

| **Hsc82** | **Hsp82** | **Hsp90alpha** | **Mutation** | **Class** | **Phenotype** |
| --- | --- | --- | --- | --- | --- |
| R46 | R46 | R60 | -G | Loading | Reduced interaction with Hsp70. Hypersensitivity to Hsp90 inhibitors. Impaired by *STI1* or *SBA1* deletion. |
| G309 | G313 | G333 | -S | Loading |  |
| K394 | K398 | K418 | -E | Loading |  |
|  |  |  |  |  |  |
| S481 | S485 | S505 | -Y | Closing | Reduced ATP-dependent interaction with Sba1 and Cpr6. Hypersensitive to Hsp90 inhibitors. Impaired by *STI1, SBA1,* or *CPR6* deletion. |
| T521 | T525 | T545 | -I | Closing |  |
| A583 | A587 | A608 | -T | Closing |  |
|  |  |  |  |  |  |
| S25 | S25 | S39 | -P | Reopening | Normal interactions with Hsp70, Sba1, and Cpr6. Not hypersensitive to Hsp90 inhibitors. Rescued by *HCH1* overexpression or *CPR6* deletion. Impaired by *HCH1* deletion or *CPR6* overexpression. |
| K102 | K102 | K116 | -E | Reopening |  |
| Q380 | Q384 | Q404 | -K | Reopening |  |
| E377 | E381 | E401 | -K | Reopening |  |
| L379 | L383 | L403 | -S | Reopening |  |
| M116 | M116 | M130 | -I |  | Impaired by *HCH1* deletion |
