# Supplementary material for: Hsp90 mutants with distinct defects provide novel insights into cochaperone regulation of the folding cycle
Source: PLoS Genet. 2023 May 25;19(5):e1010772. doi: 10.1371/journal.pgen.1010772 (PMC10246838; doi:10.1371/journal.pgen.1010772)
Supplement: S2 Table — All are isogenic to W303. (DOCX) [file pgen.1010772.s002.docx]

**S2 Table. Strains used in this study. All are isogenic to W303.**

| JJ762 | *MAT*a *ade2-1 ura3-1 leu2-3,112 trp1-1 met2-Δ1 his3-11,15 lys2-Δ2* | [1] |
| --- | --- | --- |
| JJ816 | *MAT*a *hsp82::LEU2 hsc82::LEU2/ Yep24-HSP82* | [1] |
| JJ95 | *MAT*a *hsp82:LEU2 hsc82::LEU2/ Yep24-HSP82 aha1::kan^r^* | [2] |
| JJ110 | *MAT****α*** *hsp82:LEU2 hsc82::LEU2/ Yep24-HSP82 cpr6::kan^r^* | [2] |
| JJ111 | *MAT*a *hsp82:LEU2 hsc82::LEU2/ Yep24-HSP82 hch1::kan^r^* | [2] |
| JJ146 | *MAT*a *hsp82:LEU2 hsc82::LEU2/ Yep24-HSP82 tah1::kan^r^* | [2] |
| JJ464 | *MAT****α*** *hsp82:LEU2 hsc82::LEU2/ Yep24-HSP82 tah1::kan^r^* | [2] |
| JJ94 | *MAT*a *hsp82:LEU2 hsc82::LEU2/ Yep24-HSP82 sba1::kan^r^* | [2] |
| JJ71 | *MAT***α** *hsp82:LEU2 hsc82::LEU2/ Yep24-HSP82 sgt2::kan^r^* | [3] |
| JJ833 | *MAT***α** *hsp82:LEU2 hsc82::LEU2/ Yep24-HSP82 sti1::MET2* | [4] |
| JJ1480 | *MAT****α*** *ssa1:HIS3 ssa2::TRP1 ssa3::LYS2* | This study |
| JJ623 | *MAT*a *sti1::MET2* | [1] |
| GRS4 | *hsp82::LEU2 hsc82::LEU2/ GAL1-HSP82* | [5] |
| JJ149 | *MAT*a *hsp82:LEU2 hsc82::LEU2/ Yep24-HSP82 cpr7::kan^r^* | [2] |

**Reference**

1. Flom G, Weekes J, Williams JJ, Johnson JL. Effect of mutation of the tetratricopeptide repeat and aspartate-proline 2 domains of Sti1 on Hsp90 signaling and interaction in Saccharomyces cerevisiae. Genetics. 2006;172(1):41-51. PubMed PMID: 16219779.

2. Zuehlke AD, Johnson JL. Chaperoning the Chaperone: A Role for the Co-chaperone Cpr7 in Modulating Hsp90 Function in Saccharomyces cerevisiae. Genetics. 2012;191:805-14. Epub 2012/04/17. doi: genetics.112.140319 [pii]

10.1534/genetics.112.140319. PubMed PMID: 22505624.

3. Flom GA, Langner E, Johnson JL. Identification of an Hsp90 mutation that selectively disrupts cAMP/PKA signaling in Saccharomyces cerevisiae. Curr Genet. 2012;58(3):149-63. Epub 2012/03/31. doi: 10.1007/s00294-012-0373-7. PubMed PMID: 22461145.

4. Flom G, Behal RH, Rosen L, Cole DG, Johnson JL. Definition of the minimal fragments of Sti1 required for dimerization, interaction with Hsp70 and Hsp90 and in vivo functions. Biochem J. 2007;404(1):159-67. PubMed PMID: 17300223.

5. Picard D, Khursheed B, Garabedian MJ, Fortin MG, Lindquist S, Yamamoto KR. Reduced levels of hsp90 compromise steroid receptor action in vivo. Nature. 1990;348(6297):166-8. Epub 1990/11/08. doi: 10.1038/348166a0. PubMed PMID: 2234079.
