# Supplementary figures and images for: Hsp90 mutants with distinct defects provide novel insights into cochaperone regulation of the folding cycle
Source: PLoS Genet. 2023 May 25;19(5):e1010772. doi: 10.1371/journal.pgen.1010772 (PMC10246838; doi:10.1371/journal.pgen.1010772)

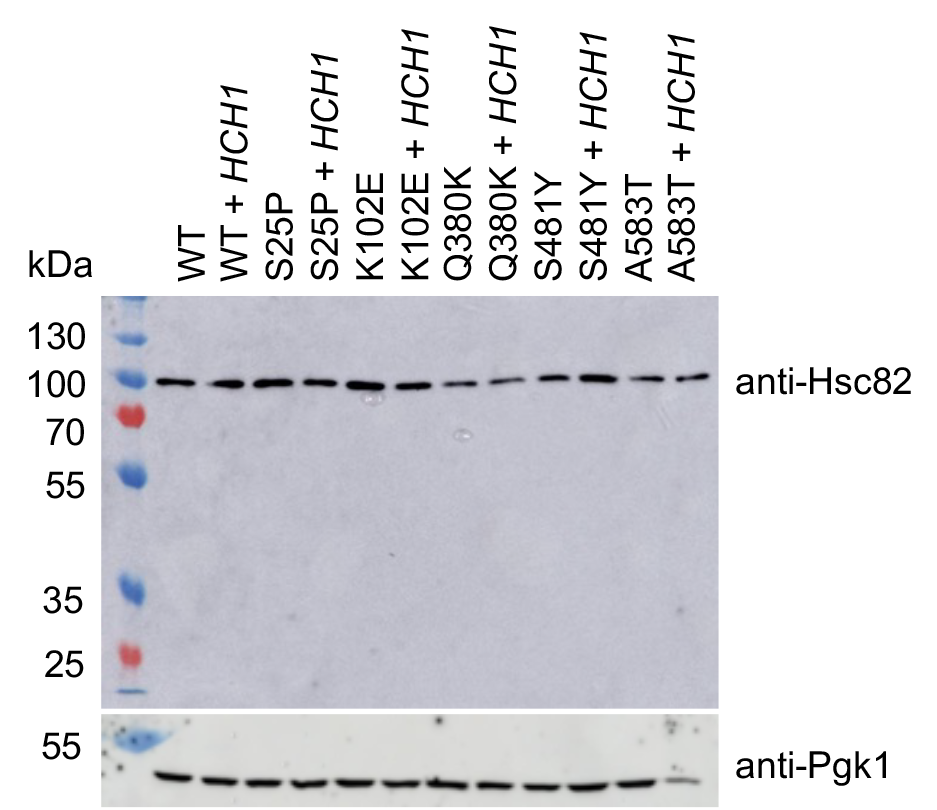

Supplement: S1 Fig — Extracts from cells expressing WT HSC82 or indicated hsc82 mutant were separated by SDS-PAGE and immunoblotted with a polyclonal antisera specific for Hsc82/Hsp82. R46G and G309S were not included due to the severe growth defect in the presence of HCH1. An antibody against Pgk1 was used as a loading control (Invitrogen catalog #459250). (TIF) [file pgen.1010772.s003.tif]

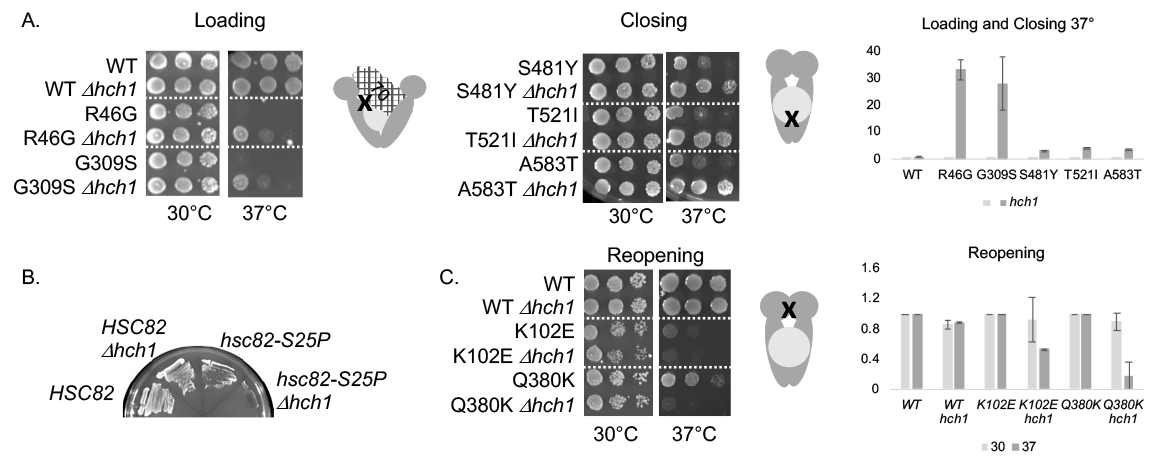

Supplement: S2 Fig — His-Hsc82 mutants were transformed into isogenic hsc82hsp82/YEp24-HSP82 (JJ816) or hch1hsc82hsp82/YEp24-HSP82 (JJ111) strains. A and C. Transformants were grown in the presence of 5-FOA to cure the YEp24-HSP82 plasmid, grown overnight at 30°C, serially diluted 10-fold, and plated on YPD and grown for two days at the indicated temperature. Right. Quantification of replicate growth assays. B. Transformants expressing wild-type HSC82 or hsc82-S25P were grown in the presence of 5-FOA for 3 days. (TIF) [file pgen.1010772.s004.tif]

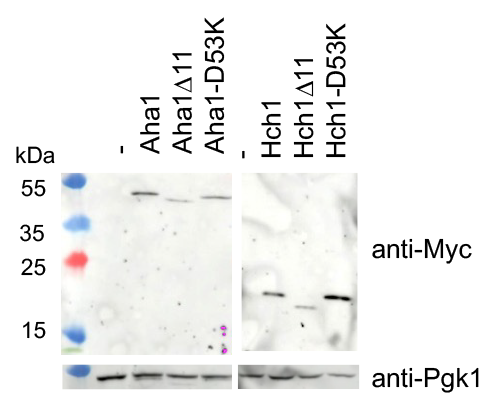

Supplement: S3 Fig — Plasmids p41KanTEF-Hch1-myc and p41KanTEF-Aha1-myc, or the empty vector (p41KanTEF) were transformed into the wildtype strain JJ762. Cell extracts were separated by SDS-PAGE and immunoblotted with a monoclonal antibody specific for Myc (Invitrogen MAI-21316). An antibody against Pgk1 (Invitrogen 459250) was used as a loading control. (TIF) [file pgen.1010772.s005.tif]

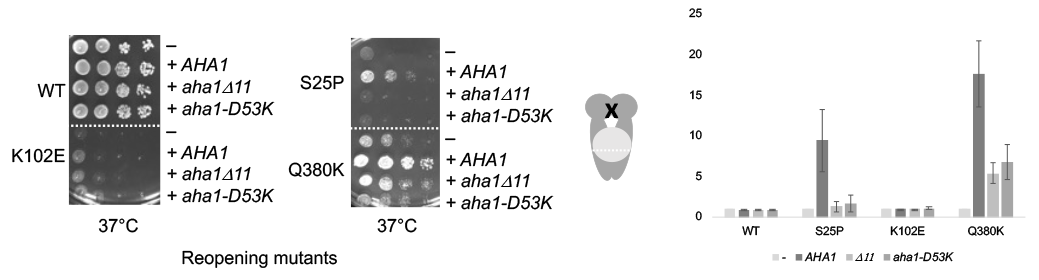

Supplement: S4 Fig — As in Fig 2, except that cells were also transformed with a plasmid overexpressing WT AHA1 (p41KanTEF-Aha1-myc, +AHA1) or plasmids that delete the NxNNWHW of Aha1(aha1-Δ11) or containing the D53K alteration (aha1-D53K). Cells were then grown overnight at 30°C, serially diluted 10-fold, and plated on selective media (YPD + G418) and grown for two days at the indicated temperature. Right. Quantification of replicate growth assays (TIF) [file pgen.1010772.s006.tif]

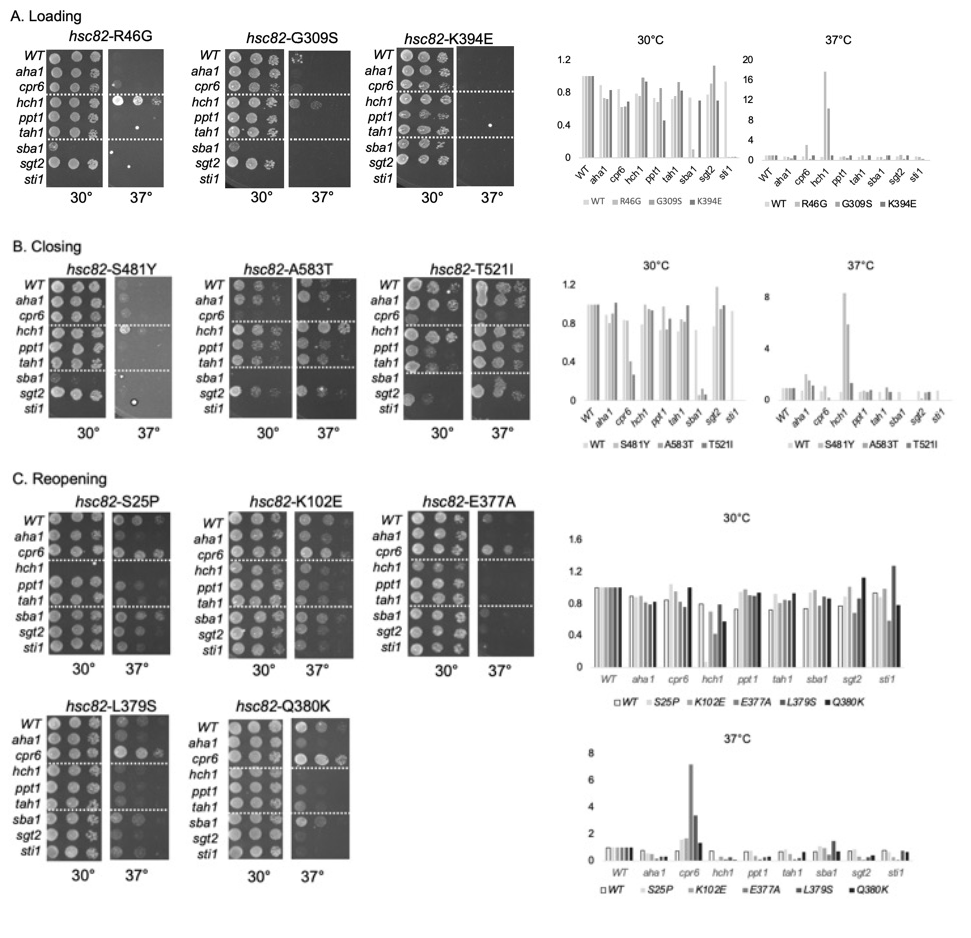

Supplement: S5 Fig — As in Fig 5, Plasmids expressing indicated WT or mutant forms of Hsc82 were expressed in isogenic hsc82hsp82 strains that do not contain deletion of any cochaperones (WT) or contain individual deletion of the cochaperone listed. Left. Cells were grown overnight at 30°C, serially diluted 10-fold, plated on rich media (YPD) and grown for two days at the indicated temperature. If no growth is shown on the 30° plate (such as with hsc82-R46G in the sti1 strain), the cells were inviable. A. Loading mutants. B. Closing mutants. C. Reopening mutants. Right. Quantification of growth assays. (TIF) [file pgen.1010772.s007.tif]

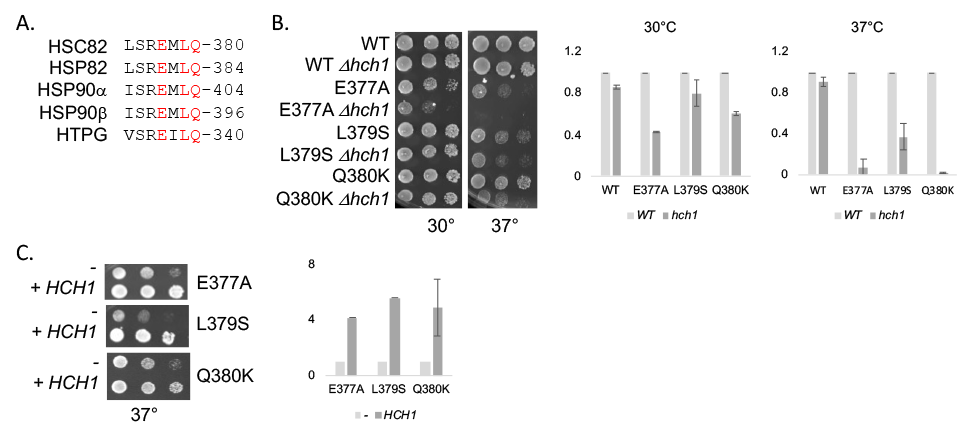

Supplement: S6 Fig — A. Conservation of residues in catalytic loop. Accession numbers used: S.cerevisiae Hsc82 KZV09036.1; S. cerevisiae Hsp82 NP_015084.1; Homo sapiens Hsp90 alpha (AAI21063.1); Homo sapiens Hsp90 beta (NP_031381.2); E. coli HtpG (NP_415006.1). B. His-Hsc82 mutants were transformed into isogenic hsc82hsp82/YEp24-HSP82 (JJ816) or hch1hsc82hsp82/YEp24-HSP82 (JJ111) strains. Transformants were grown in the presence of 5-FOA to cure the YEp24-HSP82 plasmid, grown overnight at 30°C, serially diluted 10-fold, and plated on YPD and grown for two days at the indicated temperature. C. Cells expressing the indicated mutant were transformed with empty vector (pRS426, -) or a plasmid overexpressing HCH1 (pRS426-HCH1, + HCH1). Cells were grown overnight at 30°C, serially diluted 10-fold, plated on selective media and grown for two days at the indicated temperature. Right. Quantification of replicate growth assays (TIF) [file pgen.1010772.s008.tif]

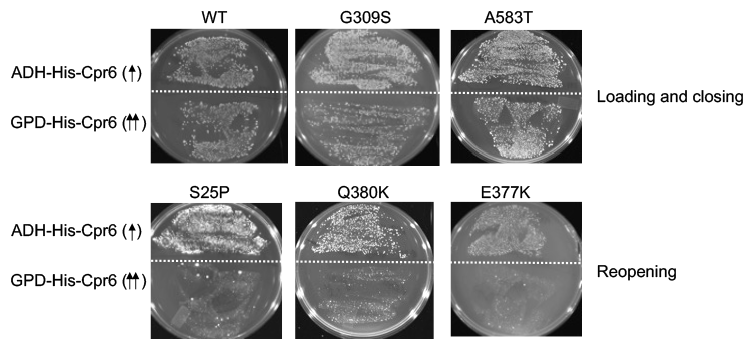

Supplement: S7 Fig — Hsc82 mutants were expressed in strain JJ816 (hsc82hsp82). Strains were transformed with plasmids pRS416ADH-His-CPR6 (top) or pRS416GPD-His-CPR6 (bottom). Transformants were grown for up to four days at 30°. Extremely small colonies are visible in cells expressing the combination of reopening mutants and GPD-His-CPR6. (TIF) [file pgen.1010772.s009.tif]

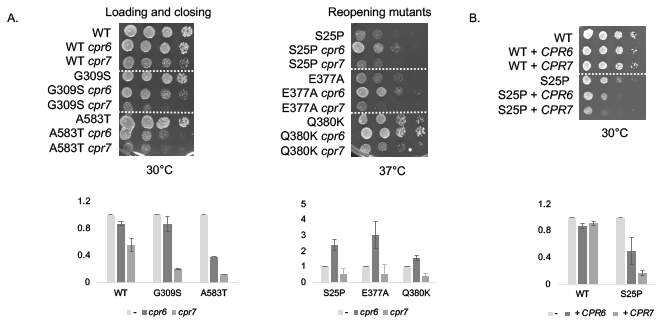

Supplement: S8 Fig — A. Hsc82 mutants were expressed in strain JJ816 (hsc82hsp82), JJ110 (cpr6hs82hsp82) or JJ149 (cpr7hsc82hsp82). Transformants were grown in the presence of 5-FOA to cure the YEp24-HSP82 plasmid, grown overnight at 30°C, serially diluted 10-fold, and plated on YPD and grown for two days at the indicated temperature. B. Hsc82 mutants expressed in strain JJ816 (hsc82hsp82), were transformed with empty vector, pRS416ADH-His-CPR6 or pRS416GPD-His-CPR7. Cells were serially diluted 10-fold, and plated on selective media and grown for two days at 30°C. Below. Quantification of replicate growth assays (TIF) [file pgen.1010772.s010.tif]

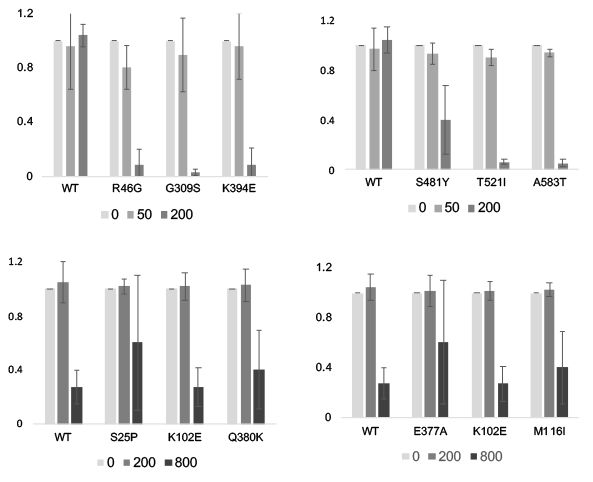

Supplement: S9 Fig — Strains were grown in YPD media and 10-fold serial dilutions were prepared and placed on agar plates with or without the indicated concentration of NVP-AUY922 where indicated and grown for 48 hours at the indicated temperatures. (TIF) [file pgen.1010772.s011.tif]
